# Diagnostic utility of movement disorder society criteria for multiple system atrophy

**DOI:** 10.3389/fnagi.2023.1200563

**Published:** 2023-06-15

**Authors:** Lingyu Zhang, Yanbing Hou, Qianqian Wei, Ruwei Ou, Kuncheng Liu, Junyu Lin, Tianmi Yang, Yi Xiao, Bi Zhao, Huifang Shang

**Affiliations:** Laboratory of Neurodegenerative Disorders, Department of Neurology, Rare Diseases Center, West China Hospital, Sichuan University, Chengdu, China

**Keywords:** multiple system atrophy, diagnostic criteria, sensitivity, specificity, cohort study

## Abstract

**Background:**

The 2008 criteria for the diagnosis of multiple system atrophy (MSA) has been widely used for more than 10 years, but the sensitivity is low, particularly for patients in the early stage. Recently, a new MSA diagnostic criteria was developed.

**Objective:**

The objective of the study was to assess and compare the diagnostic utility of the new movement disorder society (MDS) MSA criteria with the 2008 MSA criteria.

**Methods:**

This study included patients diagnosed with MSA between January 2016 and October 2021. All patients underwent regular face-to-face or telephonic follow-ups every year until October 2022. A total of 587 patients (309 males and 278 females) were retrospectively reviewed to compare the diagnostic accuracy of the MDS MSA criteria to that of the 2008 MSA criteria (determined by the proportion of patients categorized as established or probable MSA). Autopsy is the gold standard diagnosis of MSA, which is not available in clinical practice. Thus, we applied the 2008 MSA criteria at the last review as the reference standard.

**Results:**

The sensitivity of the MDS MSA criteria (93.2%, 95% CI = 90.5–95.2%) was significantly higher than that of the 2008 MSA criteria (83.5%, 95% CI = 79.8–86.6%) (*P* < 0.001). Additionally, the sensitivity of the MDS MSA criteria was maintained robustly across different subgroups, defined by diagnostic subtype, disease duration, and the type of symptom[s] at onset. Importantly, the specificities were not significantly different between the MDS MSA criteria and the 2008 MSA criteria (*P* > 0.05).

**Conclusion:**

The present study demonstrated that the MDS MSA criteria exhibited good diagnostic utility for MSA. The new MDS MSA criteria should be considered as a useful diagnostic tool for clinical practice and future therapeutic trials.

## Introduction

The motor and non-motor symptoms of patients with multiple system atrophy (MSA) progress rapidly (Pérez-Soriano et al., 2021; Zhang et al., 2022a,b), and confinement to a wheelchair occurs a short time after disease onset (Zhang et al., 2023), resulting in a shortened survival period (Low et al., 2015; Zhang et al., 2018). However, the diagnosis of MSA meets a major challenge in view of the clinical heterogeneity. The first consensus conference on the diagnosis of MSA, held in 1998, mandated three levels of diagnostic certainty—possible, probable, and definite MSA—with the diagnosis of definite MSA requiring autopsy confirmation (Gilman et al., 1999). To improve to accuracy in the diagnosis of MSA, the second consensus criteria for the diagnosis of MSA was developed in 2008 (Gilman et al., 2008), which has been widely used for more than 10 years. However, the sensitivity of MSA diagnosis at the first clinic visit was low (41% for possible and 18% for probable MSA) (Osaki et al., 2009), particularly in the early stages of MSA, resulting in delays in diagnosis and recruitment into clinical trials. Additionally, recent clinicopathological studies showed that only 62–79% of the patients with a clinical diagnosis of MSA met the pathological diagnostic criteria, and dementia with Lewy bodies (DLB), progressive supranuclear palsy (PSP), and Parkinson’s disease (PD) were the most common diseases to masquerade as MSA (Koga et al., 2015; Miki et al., 2019).

To address these potential limitations and enhance the sensitivity of early diagnosis of MSA, a movement disorder society (MDS) MSA Criteria Revision Task Force was convened to develop novel MSA diagnostic criteria (Wenning et al., 2022). The MDS criteria for the diagnosis of MSA classifies four levels of diagnostic certainty: neuropathologically established MSA, clinically established MSA, clinically probable MSA, and a new category named possible prodromal MSA (Wenning et al., 2022). The new components of the MDS MSA criteria include (Wenning et al., 2022): (1) the ≥20/10 mmHg blood pressure (BP) drop being replaced with the ≥30/15 mmHg BP drop; (2) a cutoff at >100 mL post-void residual (PVR) volume being added in the clinically established diagnosis of MSA; (3) both clinically established and clinically probable MSA requiring supportive motor or non-motor features; (4) a new research category of possible prodromal MSA. A recent study compared the 2008 MSA diagnostic criteria with the MDS criteria of MSA on 73 patients with MSA, finding a high degree of agreement (Sun et al., 2023). However, they did not compare the sensitivity and specificity of the two diagnostic criteria and the sample size was small.

Consequently, we aimed to assess the diagnostic utility of the new MDS MSA diagnostic criteria in a large clinical MSA cohort, as compared with the 2008 MSA diagnostic criteria.

## Materials and methods

Patients with suspected MSA were prospectively and consecutively enrolled into a prospective MSA cohort study from the Department of Neurology, West China Hospital of Sichuan University, between January 2016 and October 2021. All patients underwent regular face-to-face or telephonic follow-ups every year from January 2017 to October 2022 with a median follow-up period of 3 years. Exclusion criteria included a lack of clinical and imaging data and loss of follow-up after enrolling into the cohort. To exclude common forms of spinocerebellar ataxia (SCA), we performed screening for *SCA* genes including *SCA1, SCA2, SCA3, SCA6*, and *SCA7*. Patients enrolled in the current study were also subjected to brain MRI scans in our hospital.

Clinical information, including age, sex, age of onset, and disease duration was recorded at the initial clinic assessment. Patients with MSA were categorized into two subtypes, MSA-P and MSA-C, according to the predominant parkinsonian features or cerebellar ataxia, respectively. Disease severity was evaluated using the Unified MSA Rating Scale (UMSARS) with part I (activities of daily living, ADL), part II (motor examination), part III (autonomic examination), and part IV (global disability) (Wenning et al., 2004). The total UMSARS score is the sum of parts I and II. Blood pressure and heart rate (HR) measurements were taken at 1-, 3-, 5-, and 10-min intervals in an upright position and were compared with the measurements taken in a supine position (Pavy-Le Traon et al., 2016), and the ΔHR/Δsystolic BP (SBP) ratio were calculated.

All patients were classified as possible or probable MSA according to the 2008 MSA criteria at the initial visit. They were retrospectively reviewed according to the MDS MSA diagnostic criteria by two physicians independently. The final diagnosis was based on the 2008 MSA criteria at the last review. The diagnosis made based on the 2008 or MDS criteria was then compared with the final diagnosis as a reference standard. The calculation of the diagnostic sensitivities and specificities of the 2008 and MDS MSA diagnostic criteria were according to the clinically established or clinically probable MSA diagnostic categories as a positive finding.

The study design was approved by the Ethics Committee of the West China Hospital of Sichuan University. Informed written consent was obtained from all participants.

### Statistical analysis

The sensitivities and specificities of the two criteria for the diagnosis of MSA were the primary outcome measures. The secondary outcome measures consisted of diagnostic utility in MSA subgroups defined by the diagnostic subtype (MSA-P vs. MSA-C), disease duration (<3 years or ≥3 years at the time of initial visit), and the type of symptom[s] at onset (motor symptom or autonomic symptom). Sensitivity and specificity were calculated for each criterion.

For the MDS criteria:

**Table:** 

	Established/probable MSA (MDS, first visit)	Non-MSA (MDS, first visit)
Probable MSA (final diagnosis)	True Positive (TP)	False Negative (FN)
Possible MSA (final diagnosis)	False Positive (FP)	True Negative (TN)

For the 2008 criteria:

**Table:** 

	Probable MSA (2008, first visit)	Possible MSA (2008, first visit)
Probable MSA (final diagnosis)	TP	FN
Possible MSA (final diagnosis)	FP	TN


$$\mathrm{Sensitivity}=\frac{T\,P}{T\,P+F\,N}\times 100\%,\mathrm{Specificity}=\frac{T\,N}{T\,N+F\,P}\times 100\%.$$

The McNemar test was utilized to assess the differences between the two different criteria. The data analyses were performed using the IBM SPSS Statistics software (version 26.0). A *p*-value < 0.05 was considered statistically significant.

## Results

**FIGURE 1:**
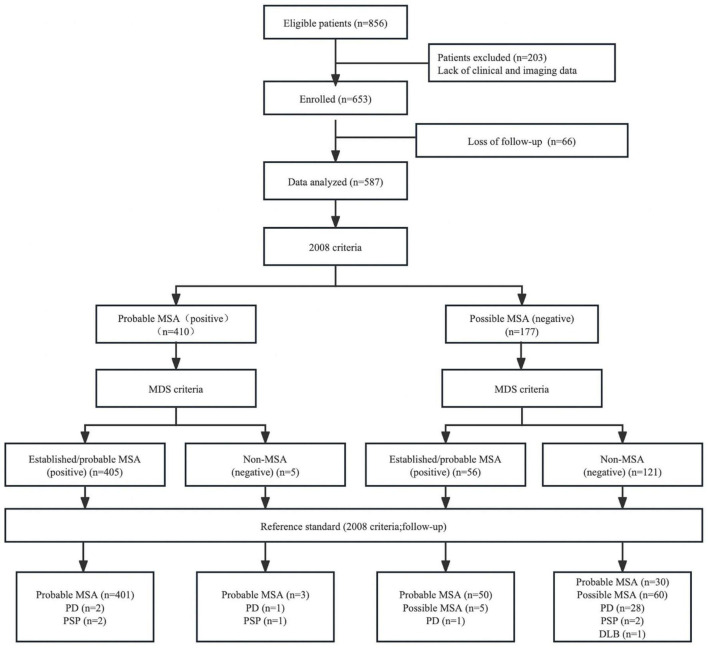
Flow diagram of the study. MSA, multiple system atrophy; MDS, movement disorders society; PD, Parkinson’s disease; PSP, progressive supranuclear palsy; DLB, dementia with Lewy bodies.

In total, 587 patients (309 males, 278 females, mean age = 61.09 ± 8.87 years) were enrolled in the current study. The mean disease duration from symptom onset in patients with MSA at the initial assessment was 2.56 ± 1.56 years. Approximately 80% of the patients had atrophy of the cerebellum, 63.2% had atrophy of the middle cerebellar peduncle, 45.0% had atrophy of the putamen, 42.0% had atrophy of the pons, and 23.2% had a “hot cross bun” sign. Finally, 484 patients were diagnosed with probable MSA (249 males, 235 females, mean age = 60.92 ± 8.72 years) after regular follow-up. One hundred and three patients were diagnosed with possible or non-MSA disease, including 32 PD, five PSP, one DLB, and 65 possible MSA (Figure 1 and Table 1).

**TABLE 1:** Demographic and clinical details of the study population.

Variables	Total	Probable MSA	Possible- or non-MSA disease
Number	587	484	103
Age	61.09 ± 8.87	60.92 ± 8.72	61.91 ± 9.59
Age of onset	58.50 ± 8.80	58.27 ± 8.62	59.54 ± 9.57
Sex (male/female)	309/278	249/235	60/43
Diagnosis subtype (MSA-P/MSA-C)	303/284	230/254	73/30
Onset of symptom (motor/autonomic)	405/182	322/162	83/20
Disease duration	2.56 ± 1.56	2.63 ± 1.55	2.24 ± 1.56
UMSARS-I score	16.32 ± 7.56	17.60 ± 7.37	10.31 ± 5.16
UMSARS-II score	18.71 ± 7.82	19.74 ± 7.79	13.86 ± 5.99
UMSARS-IV score	2.27 ± 1.07	2.40 ± 1.08	1.64 ± 0.77
UMSARS total score	35.03 ± 14.59	37.34 ± 14.36	24.17 ± 10.13
**MRI makers**			
Atrophy of putamen	263 (45.0%)	249 (51.4%)	14 (13.6%)
Atrophy of middle cerebellar peduncle	371 (63.2%)	345 (71.3%)	26 (25.2%)
Atrophy of pons	247 (42.0%)	236 (48.8%)	11 (10.7%)
Atrophy of cerebellum	468 (79.7%)	410 (84.7%)	58 (56.3%)
“Hot cross bun” sign	136 (23.2%)	134 (27.7%)	2 (1.9%)

MSA, multiple system atrophy; MSA-P, multiple system atrophy with predominant parkinsonism; MSA-C, multiple system atrophy with predominant cerebellar ataxia; UMSARS, Unified Multiple System Atrophy Rating Scale; MRI, magnetic resonance imaging.

At the first visit, 12 patients with a diagnosis of possible MSA according to the 2008 MSA criteria met the diagnosis of clinically established MSA according to the MDS criteria due to orthostatic hypotension (OH) definition (nine patients), PVR definition (two patients), or both (one patient). Meanwhile, 44 patients with a diagnosis of possible MSA met the diagnosis of clinically probable MSA according to the MDS criteria due to OH definition (30 patients), PVR definition (seven patients), or both (seven patients).

The diagnostic accuracy of each type of MSA diagnostic criteria are shown in Table 2. The diagnostic sensitivities to the established or probable MSA were 83.5% (95% CI = 79.8–86.6%) and 93.2% (95% CI = 90.5–95.2%) for the 2008 MSA criteria and MDS MSA criteria, respectively, with a significant difference between them (*P* < 0.001). Additionally, the specificities were not significantly different between the 2008 MSA criteria and MDS MSA criteria [94.2% (95% CI = 87.2–97.6%) vs. 90.3% (95% CI = 82.5–95.0%), respectively, *P* = 0.289]. The sensitivity of the MDS MSA criteria was improved, but the specificity was not significantly reduced.

**TABLE 2:** Diagnostic accuracy of MSA criteria.

	2008 criteria (95% CI)	MDS criteria (95% CI)	*P*-value
**Total patients**			
Sensitivity, %	83.5 (79.8–86.6)	93.2 (90.5–95.2)	<0.001*
Specificity, %	94.2 (87.2–97.6)	90.3 (82.5–95.0)	0.289
**MSA-P**			
Sensitivity, %	84.8 (79.3–89.0)	95.2 (91.3–97.5)	<0.001*
Specificity, %	93.1 (84.1–97.5)	91.8 (82.4–96.6)	1.000
**MSA-C**			
Sensitivity, %	82.3 (76.9–86.7)	91.3 (87.0–94.4)	<0.001*
Specificity, %	96.7 (80.9–99.8)	86.7 (68.4–95.6)	0.250
**Disease duration <3 years**			
Sensitivity, %	79.4 (74.3–83.6)	90.3 (86.3–93.3)	<0.001*
Specificity, %	97.1 (89.0–99.5)	94.2 (85.1–98.1)	0.625
**Disease duration ≥3 years**			
Sensitivity, %	90.8 (85.2–94.5)	98.3 (94.6–99.6)	<0.001*
Specificity, %	88.2 (71.6–96.2)	82.4 (64.8–92.6)	0.625
**Motor symptom onset**			
Sensitivity, %	81.7 (76.9–85.7)	92.5 (89.0–95.1)	<0.001*
Specificity, %	94.0 (85.9–97.8)	89.2 (80.0–94.6)	0.289
**Autonomic symptom onset**			
Sensitivity, %	87.0 (80.6–91.6)	94.4 (89.4–97.3)	<0.001*
Specificity, %	95.0 (73.1–99.7)	95.0 (73.1–99.7)	1.000

MSA, multiple system atrophy; MSA-P, multiple system atrophy with predominant parkinsonism; MSA-C, multiple system atrophy with predominant cerebellar ataxia; MDS, movement disorder society. * Significant difference.

Subgroup analyses are also shown in Table 2. In the MSA-P subgroup, the diagnostic sensitivity of the MDS MSA criteria was significantly higher than that of the 2008 MSA criteria (95.2% vs. 84.8%, respectively, *P* < 0.001). Meanwhile, in the MSA-C subgroup, the diagnostic sensitivity of the MDS MSA criteria was significantly higher than that of the 2008 MSA criteria (91.3% vs. 82.3%, respectively, *P* < 0.001). Of note, the specificities were also not significantly different between the two criteria in both subgroups (*P* > 0.05). When performing subgroup analysis according to the disease duration, the sensitivity of the MDS MSA criteria was significantly higher when compared with the 2008 MSA criteria in both subgroups (*P* < 0.001). Additionally, the sensitivity of the MDS MSA criteria was observed to be similar in patients with motor and autonomic symptom onset, being significantly higher than the 2008 MSA criteria (*P* < 0.001). The specificities were not significantly different between the two criteria in both subgroups (*P* > 0.05).

## Discussion

We highlighted that the sensitivity of the MDS MSA criteria was significantly higher than that of the 2008 MSA criteria, and the sensitivity remained robust across subgroups. Additionally, the specificity of the MDS MSA criteria were shown to be comparable to the 2008 MSA criteria.

The clinical diagnosis of MSA has relied on identification of autonomic failure along with poorly levodopa-responsive parkinsonism or a cerebellar syndrome in the absence of postmortem confirmation. In the 2008 criteria, definite MSA needs the neuropathologic findings of widespread and abundant α-synuclein–positive glial cytoplasmic inclusions in association with neurodegenerative changes in striatonigral or olivopontocerebellar structures (Gilman et al., 2008). The diagnosis of probable MSA requires autonomic failure involving urinary incontinence or OH (the ≥30/15 mmHg BP drop) along with poorly levodopa-responsive parkinsonism or a cerebellar syndrome. Meanwhile, possible MSA requires one or more features suggestive of autonomic dysfunction associated with parkinsonism or a cerebellar syndrome, plus one of the additional features. However, the sensitivity of the 2008 MSA criteria was low, particularly at the early stage of the disease, with common misdiagnoses including DLB, PSP, and PD (Miki et al., 2019). To improve diagnostic accuracy in clinical practice (particularly at the early stage of MSA) and the recruitment in clinical trials of disease-modifying treatments, a new MDS MSA criteria was developed (Wenning et al., 2022).

There are several improvements to the 2008 MSA criteria that should be discussed. The MDS MSA criteria includes four levels of diagnostic accuracy: neuropathologically established MSA, clinically established MSA, clinically probable MSA, and possible prodromal MSA. OH is determined by consensus as a drop of systolic BP (SBP) >20 mmHg and/or of diastolic BP (DBP) >10 mmHg within 3 min in an upright position (The Consensus Committee of the American Autonomic Society and the American Academy of Neurology, 1996). The 2008 criteria for probable MSA requires a higher cut-off value to diagnose neurogenic OH (the ≥30/15 mmHg BP drop). The classical ≥20/10 mmHg BP drop has better sensitivity for discriminating MSA from PD compared to the ≥30/15 mmHg BP drop with similar specificity (≥20/10 mmHg, sensitivity: 46% and specificity: 73%; ≥30/15 mmHg, sensitivity: 28% and specificity: 80%) (Fanciulli et al., 2019). Performing orthostatic testing over 10 min allows an additional 20% of patients with MSA with significant OH to be detected (Pavy-Le Traon et al., 2016). A prolonged 10 min neurogenic OH was defined as a feature of clinically probable MSA in the MDS MSA criteria. According to the MDS MSA criteria, the ≥20/10 mmHg BP drop replaced the ≥30/15 mmHg BP drop criterion of the 2008 MSA criteria, which increased the sensitivity of the diagnosis of MSA. We found that 10 patients with a diagnosis of possible MSA according to the 2008 MSA criteria met the diagnosis of clinically established MSA according to the MDS criteria due to OH definition. Meanwhile, 37 patients with a diagnosis of possible MSA met the diagnosis of clinically probable MSA according to the MDS criteria due to OH definition.

Urinary dysfunction often occurs early in patients with MSA during the course of the disease (Ito et al., 2006). The average PVR volume was 71 mL in the first year, increasing to 170 mL in the fifth year (Ito et al., 2006). Incomplete voiding (>100 ml of residual volume) was found in 11 patients (55%) with MSA but only in one patient (5%) with PD (Hahn and Ebersbach, 2005). The positive predictive value of PVR volume >100 ml was 91.6% for a diagnosis of MSA (Hahn and Ebersbach, 2005). Urinary incontinence is one of the core features in the diagnosis of probable MSA based on the 2008 MSA criteria (Gilman et al., 2008). However, urinary retention (PVR volume >100 ml) has extremely higher specificity for discriminating MSA from PD compared to urinary incontinence (urinary retention, sensitivity: 34% and specificity: 95%; urinary incontinence, sensitivity: 48% and specificity: 34%) (Fanciulli et al., 2019). A clinically established diagnosis of MSA requires a cutoff at >100 mL PVR volume to secure high specificity, while a finding of PVR volume <100 ml is sufficient for the diagnosis of clinically probable MSA. We found that three patients with a diagnosis of possible MSA according to the 2008 MSA criteria met the diagnosis of clinically established MSA according to the MDS criteria due to PVR definition. Meanwhile, 14 patients with a diagnosis of possible MSA met the diagnosis of clinically probable MSA according to the MDS criteria due to PVR definition.

A diagnosis of clinically established MSA requires at least one structural brain MRI marker of atrophy or diffusivity changes in the infratentorial or putamen regions (Wenning et al., 2022). Most MSA neuroimaging studies have been concentrated on the gray matter atrophy pattern shown on structural T1 MRI and signal changes seen on T2, FLAIR, and T2* MRI (Chelban et al., 2019). While structural brain MRI abnormalities show high specificity for distinguishing MSA from PD, their sensitivity remains limited, particularly in the early stages (Feng et al., 2015; Krismer et al., 2019). Differences in putamen diffusivity are highly accurate for discriminating patients with MSA-P from PD (sensitivity, 90%; specificity, 93%) (Pellecchia et al., 2020). Additionally, at least two supportive clinical (either motor or non-motor) features (previously termed “red flags”) are necessary for diagnosing clinically established MSA, and only one for clinically probable MSA.

To our knowledge, this is the largest diagnostic study of MSA that assessed the diagnostic utility of the MDS MSA criteria compared with the 2008 MSA criteria. As expected, the MDS MSA criteria exhibited significantly higher sensitivity compared to the 2008 MSA criteria, considering unexplained voiding difficulties with PVR volume and the ≥20/10 mmHg BP drop for the diagnosis of probable/established MSA. Additionally, the specificity of the MDS MSA criteria remained high. Diagnostic subtype, disease duration, and symptoms of onset did not obviously impact on the sensitivity of the MDS MSA criteria. Specifically, the MDS MSA criteria sensitivity was significantly higher in patients with a shorter disease duration. Recently, Virameteekul et al. (2023) reported that the MDS MSA criteria demonstrated excellent diagnostic performance against neuropathological diagnosis, which supports our results. Consequently, the MDS MSA criteria may prove to be more robust in recruiting early MSA into clinical trials.

This study had several limitations. First, this study involved the retrospective application of the MDS MSA diagnostic criteria, which may have introduced some bias. Second, all patients were recruited through a tertiary referral center in western China, and further multicenter collaboration is needed to confirm our results. Third, all patients were clinically diagnosed without a postmortem diagnosis. Fourth, other parkinsonisms were not included at the beginning of the study.

## Conclusion

In conclusion, autopsy is the gold standard diagnosis of MSA but is not available in clinical practice. Thus, we applied the 2008 MSA criteria at the last review as the reference standard. This large cohort study suggests that the diagnostic sensitivity of the MDS MSA criteria is significantly higher than that of the 2008 MSA criteria. The new MDS MSA criteria should be considered as useful diagnostic tools for clinical practice and therapeutic trials.

## Data availability statement

The datasets used and/or analyzed during the current study are available from the corresponding author on reasonable request.

## Ethics statement

The studies involving human participants were reviewed and approved by The Ethics Committee of West China Hospital of Sichuan University. The patients/participants provided their written informed consent to participate in this study.

## Author contributions

LZ: conception and design of the study, statistical analysis, interpretation of data, and drafting of the manuscript. YH, QW, RO, KL, JL, TY, YX, and BZ: data collection. HS: study design, analysis, interpretation, and revision of the manuscript. All authors contributed to the article and approved the submitted version.
